# Kinesin motor proteins as host factors regulating viral infection: Mechanisms of exploitation, emerging paradigms, and therapeutic horizons

**DOI:** 10.20407/fmj.2026-014

**Published:** 2026-09-25

**Authors:** Hussein Hassan Aly

**Affiliations:** Japan Institute for Health Security, National Institute of Infectious Diseases, Department of Virology 2, Shinjuku, Tokyo, Japan

**Keywords:** Kinesin, Intracellular transport, Viral trafficking, Host factor, Antiviral therapy

## Abstract

Intracellular transport via microtubules is integral to eukaryotic cell function, and viruses have developed intricate mechanisms to exploit this system at nearly every stage of their replication cycle. The mammalian kinesin superfamily, consisting of 14 distinct families, enables anterograde transport that is used by both DNA and RNA viruses. This review provides a comprehensive analysis of how a wide range of viral pathogens—including herpesviruses, human immunodeficiency virus type 1, vaccinia virus, hepatitis B virus (HBV), influenza virus, and severe acute respiratory syndrome coronavirus—interact with specific kinesin family members to promote viral entry, genome trafficking, organization of replication compartments, assembly, and egress. Recent findings underscore the role of the kinesin-4 family member KIF4A as a novel host factor for HBV. The therapeutic potential of modulating the surface expression of the HBV-entry receptor (NTCP) is discussed, either by suppressing KIF4 expression—necessary for anti-retrograde transport of NTCP to the cell surface—or by disrupting the NTCP–KIF4 interaction. Additionally, emerging pharmacological approaches targeting kinesin-dependent transport are evaluated. By establishing a unified framework that connects motor protein biology to virology, this review identifies key knowledge gaps and outlines future research directions for the development of broad-spectrum, host-targeted, antiviral therapies.

## Introduction

For a virus to successfully complete a replication cycle inside a host cell, it must move through the complex and hostile environment of the cell cytoplasm. The cytoplasm presents a formidable physical barrier—molecular crowding limits free diffusion of particles larger than approximately 500 kDa,^1^ while the actin meshwork and organelle architecture add spatial limitations. In addition, innate immune sensors detect and eliminate foreign nucleic acids and protein complexes. To address these challenges, nearly all animal viruses studied to date have developed strategies to use the host cell’s microtubule (MT)-dependent transport system.^2,3^ MTs are dynamic polymers composed of α/β-tubulin heterodimers that radiate from the centrosome (or other MT-organizing centers) toward the edge of the cell. This creates a polarized highway system inside the cell. Two main types of molecular motors operate on this highway: dyneins, which promote trafficking toward the nucleus (retrograde) from the minus end, and kinesins, which promote trafficking toward the periphery (anterograde) from the plus end. However, there are some important exceptions in both families.^4,5^ Although the viral exploitation of dynein for intracellular trafficking during entry has been thoroughly examined,^6,7^ the equally critical roles of kinesin motors in viral replication have received comparatively less systematic attention.

The kinesin superfamily (KIF) encompasses 45 genes in the human genome, organized into 14 families (kinesin-1 through kinesin-14) based on phylogenetic analysis of the conserved motor domain.^8^ Each family displays distinct properties with respect to processivity, velocity, cargo specificity, and subcellular localization, providing viruses with a diverse toolkit for intracellular manipulation. Beyond simple cargo transport, kinesins regulate MT dynamics (kinesin-8, kinesin-13), organize the mitotic spindle (kinesin-5, kinesin-12, kinesin-14), mediate chromosome condensation and cytokinesis (kinesin-4), and contribute to organelle positioning and membrane trafficking (kinesin-3).^5^ Viruses exploit not only the transport function but also these regulatory activities to remodel the cellular environment to their advantage.

Two mechanistically distinct modes by which viruses depend on kinesin motors are known. In the first, namely direct viral exploitation, viral proteins physically engage kinesin motors or their adaptor proteins to drive the active transport of viral components (capsids, tegument, glycoprotein vesicles, or Gag polyprotein) along MTs. The interactions of vaccinia virus (VACV) F12L protein and human immunodeficiency virus type 1 (HIV-1) Gag polyprotein with KIF4 are archetypal examples of this direct mechanism. In the second, namely indirect host dependency, viruses do not directly interact with kinesin motors but instead rely on constitutive, kinesin-mediated trafficking of host proteins—most notably, entry receptors—to enable infection. The KIF4A–NTCP axis in hepatitis B virus (HBV) exemplifies this indirect mode: KIF4A mediates the anterograde delivery of the host receptor NTCP to the hepatocyte surface as part of normal cellular physiology, and HBV passively benefits from this constitutive transport to gain access to sufficient receptor density for efficient entry. These two mechanisms are fundamentally distinct in their molecular logic and have different implications for antiviral targeting strategies, and are treated as separate categories throughout this review.

This review provides a comprehensive and systematic examination of kinesin exploitation across viral families. We integrate classical studies on herpesvirus axonal transport with recent discoveries, including the identification of KIF4A as a host factor for HBV^9^ and the development of a retinoid X receptor (RXR) agonist (S169), which suppresses the expression of KIF4A. By downregulating KIF4A, S169 reduces the transport of NTCP to the cell surface, thereby limiting NTCP-mediated entry of HBV and hepatitis delta virus (HDV). This mechanism highlights the potential utility of S169 in blocking viral entry by targeting host factors essential for infection.^10^

## Kinesin biology: Structure and regulation

### General structure and mechanochemistry

Conventional kinesin (kinesin-1/KIF5) serves as the structural paradigm for the superfamily. The active kinesin protein complex consists of two kinesin heavy chains (KIF5A, KIF5B, or KIF5C) and two kinesin light chains (KLC1–4).^11^ Each heavy chain comprises an N-terminal globular motor domain (head) that contains both the MT-binding interface and the ATP hydrolysis active site, a neck linker that undergoes conformational changes coupled to the ATPase domain, an extended coiled-coil stalk mediating heavy chain dimerization, and a C-terminal tail that interacts with KLCs and, in some cases, directly with cargo.^5^

The mechanochemical cycle of kinesin-1 involves a hand-over-hand stepping mechanism with an 8-nm step size corresponding to the tubulin dimer repeat, generating forces of approximately 6–7 pN and achieving velocities of 0.6–1.0 μm/s *in vitro*.^11^ Each step is coupled to the hydrolysis of one ATP molecule, making kinesin one of the most efficient biological motors known. The two heads operate in a coordinated, alternating manner that maintains at least one head bound to the MT at all times, ensuring high processivity (run lengths of ~1 μm).^11^

### The 14 kinesin families: Functional diversity

The 14 kinesin families display remarkable functional diversification^5,9,12–15^ (Table 1).

### Cargo recognition and regulatory mechanisms

Cargo specificity is achieved through multiple mechanisms. KLCs contain tetratricopeptide repeat (TPR) domains that recognize short linear motifs (such as W-acidic and Y-acidic motifs) in cargo adaptors.^16^ Adaptor proteins—including JIP1/2/3 (c-Jun N-terminal kinase-interacting proteins), GRIP1, Milton/TRAK1/2, and various Rab effectors—bridge motors to specific vesicular or organellar cargoes.^17,18^

Kinesin activity is regulated by autoinhibition, wherein the tail domain folds back to interact with the motor domain, preventing futile ATP hydrolysis in the absence of cargo.^19^ Cargo binding relieves autoinhibition, coupling motor activation to productive transport. Post-translational modifications of both kinesins (phosphorylation, ubiquitination) and tubulin (acetylation, detyrosination, polyglutamylation) further modulate transport efficiency and directionality. The “tubulin code” hypothesis posits that specific patterns of tubulin modification create preferred tracks for distinct motor-cargo combinations.^20^ This regulatory complexity provides multiple nodes at which viruses can intervene.

## Exploitation of kinesins across viral families

### Alphaherpesviruses: The paradigm of direct kinesin exploitation

#### 1 Biology and the transport challenge

The alphaherpesviruses, including herpes simplex virus type 1 (HSV-1), pseudorabies virus (PRV), and varicella-zoster virus (VZV), face a unique transport challenge. Following primary infection of epithelial cells, viral capsids must travel retrogradely along sensory axons—distances that can exceed 1 meter in humans—to reach neuronal cell bodies in ganglia where latency is established. Upon reactivation, newly assembled virions or subviral assemblies must traverse the same axonal distances in the anterograde direction to reach sites of recurrent disease^18,19^. This system represents a paradigmatic example of direct viral exploitation of kinesin motors, as viral tegument and membrane proteins physically engage kinesin complexes to drive anterograde transport (Figure 1).

#### 2 Anterograde transport mechanisms

The anterograde transport of alphaherpesvirus components in axons has been a subject of intense investigation and debate. Two models have been proposed: the “Married” model, in which fully assembled enveloped virions within transport vesicles are carried as single entities, and the “Separate” model, in which capsids and glycoprotein-containing vesicles are transported independently and assemble at the axon terminus.^21^ Current evidence supports aspects of both models depending on the virus and experimental system, although for HSV-1 the predominant view favors transport of assembled particles within vesicles.^21^

Kinesin-1 (KIF5) family members serve as the primary anterograde motors. The HSV-1 membrane protein pUS9 has been implicated in kinesin recruitment.^22^ For PRV, the US9 membrane protein is essential for anterograde axonal sorting, functioning together with glycoproteins gE and gI.^23^ The outer tegument protein pUL36 (VP1/2), the largest herpesvirus protein at >3,000 amino acids, directly engages the dynein–dynactin retrograde motor complex during entry and may regulate the switch to kinesin-dependent anterograde transport through its interaction with the inner tegument protein pUL37.^24,25^

More recently, kinesin-3 family members (particularly KIF1A) have been identified as contributors to HSV-1 capsid transport in axons, suggesting that alphaherpesviruses may simultaneously engage multiple kinesin families to ensure robust anterograde delivery.^26^ This motor redundancy may help explain why single-motor knockdowns often produce incomplete transport phenotypes (Figure 1).

#### 3 Regulation of transport directionality

A critical question is how viruses switch between dynein-dependent retrograde movement (during entry) and kinesin-dependent anterograde movement (during egress). The phosphorylation state of viral tegument proteins, particularly through the activity of the viral US3 kinase, has been proposed to regulate this switch by modulating interactions with motor complexes.^27^ The concept of a “molecular switch” coordinating bidirectional transport is central to understanding herpesvirus pathogenesis and has implications for other neurotropic viruses.

### Retroviridae: HIV-1

#### 1 Inward trafficking during entry

Following membrane fusion and entry into the cytoplasm, the HIV-1 reverse transcription complex and pre-integration complex must be transported to the nucleus. Dynein-mediated retrograde transport plays a dominant role in delivering the incoming complex toward the nuclear pore; however, kinesin-dependent outward movements are also observed, and the net movement represents a biased random walk favoring the perinuclear region.^28^ The viral capsid protein (CA) and the accessory protein Vpr have been implicated in recruiting MT motors, although the precise adaptor mechanisms remain incompletely defined.^28^

#### 2 Outward trafficking during assembly and budding

The chromokinesin KIF4 has been identified as a direct binding partner of the HIV-1 Gag polyprotein, facilitating its MT-dependent transport to the plasma membrane for virion assembly and release. The interaction between KIF4 and Gag was first demonstrated by Tang et al.,^29^ who showed that KIF4 binds to the matrix (MA) domain of Gag and that siRNA-mediated depletion of KIF4 significantly reduced virus-like particle release without affecting Gag protein expression levels. This finding indicated that KIF4 functions specifically in the transport of Gag to assembly sites rather than in Gag synthesis or stability. Furthermore, disruption of MTs with nocodazole phenocopied the effect of KIF4 knockdown, confirming the dependence of this transport pathway on an intact MT network. These observations established KIF4 as a critical host factor co-opted by HIV-1 to ensure efficient delivery of its major structural protein to the site of budding.^15^

### Poxviridae: Vaccinia virus

VACV provides one of the most thoroughly characterized examples of direct kinesin exploitation via molecular mimicry. VACV produces two forms of infectious particles: intracellular mature virions (IMV) and extracellular enveloped virions (EEV). The latter are critical for cell-to-cell spread and long-range dissemination.^30^

The VACV protein F12L directly interacts with kinesin-1 light chain through a canonical W-acidic motif, mimicking host cargo adaptors.^31^ This interaction is essential for the transport of intracellular enveloped virions (IEV, the intracellular precursor of EEV) from perinuclear replication factories to the cell surface. Disruption of F12L results in accumulation of IEV in the perinuclear region and a dramatic reduction in EEV release and viral spread.^31^

The A36R membrane protein of IEV is phosphorylated by Src-family kinases on tyrosine residues, creating docking sites for the adaptor proteins Nck and Grb2, which in turn activate N-WASP and the Arp2/3 complex to nucleate actin tails that propel virions through the cortical cytoskeleton after MT-dependent transport delivers them to the cell periphery.^32,33^ This two-phase transport mechanism—kinesin-dependent long-range MT transport followed by actin-based propulsion—represents an elegant example of coordinated cytoskeletal exploitation.

### Hepadnaviridae: Hepatitis B virus and the KIF4A paradigm of indirect host dependency

#### 1 KIF4A as a regulator of HBV entry

Aly’s laboratory identified the kinesin-4 family member KIF4A as a host factor that regulates the anterograde transport of the HBV entry receptor NTCP (sodium taurocholate co-transporting polypeptide) to the cell surface, thereby influencing HBV entry into hepatocytes.^9^ KIF4A is a chromokinesin with established roles in chromosome condensation, DNA damage repair, and cytokinesis. Its identification as a regulator of NTCP surface expression was unexpected and highlights the capacity of viruses to depend on host motors with functions seemingly unrelated to canonical cargo transport.

Crucially, in this system, there is no direct interaction between KIF4A and any HBV protein. Rather, KIF4A functions as a constitutive regulator of NTCP trafficking within normal hepatocyte physiology. HBV exploits this pre-existing transport pathway passively: provided that KIF4A drives NTCP to the plasma membrane as part of normal cellular homeostasis, sufficient receptor density is maintained for efficient viral binding and entry. The virus is therefore not an active participant in the transport event itself but is entirely dependent on its outcome (Figure 2).

Mechanistically, KIF4A facilitates the anterograde transport of NTCP from intracellular compartments to the plasma membrane, ensuring sufficient surface expression of the receptor to support efficient HBV binding and entry. Suppression of NTCP transport by siRNA-mediated silencing of KIF4A expression significantly reduced NTCP surface levels and, consequently, HBV entry into target cells. Importantly, this finding was further validated pharmacologically: treatment with bexarotene, an RXR agonist, similarly suppressed NTCP surface transport, leading to a significant reduction in HBV infection. This pharmacological approach is particularly noteworthy because bexarotene is an FDA-approved drug originally developed for cutaneous T-cell lymphoma, raising the possibility of drug repurposing as an antiviral strategy targeting the host transport machinery rather than viral components directly.

These findings establish a model in which HBV entry is critically dependent on kinesin-mediated delivery of its receptor to the cell surface, representing a vulnerable node in the viral life cycle that can be therapeutically targeted. Rather than a direct interaction between KIF4A and viral proteins, this mechanism reveals an indirect but essential role for kinesin motors in viral infection, whereby the virus depends on constitutive host transport pathways to maintain the availability of its entry receptor.

## Mechanisms of kinesin engagement by viruses

### Direct molecular mimicry

The most elegant mechanism of kinesin co-optation involves viral proteins that directly mimic host cargo adaptors. The VACV F12L protein provides the best-characterized example: its W-acidic motif binds to the KLC-TPR domain in a manner indistinguishable from endogenous cargo adaptors.^2,31^ This molecular mimicry allows the virus to engage the transport machinery without requiring host protein intermediaries, providing both specificity and efficiency.

Similarly, herpesvirus tegument proteins (pUL36, pUL37) may function as molecular mimics of endogenous motor adaptors, though the structural basis for their interactions with kinesin is less well defined.^24,25^ The general principle—viral structural or tegument proteins evolving binding motifs that resemble host cargo signals—is likely to be widespread.

### Direct binding to kinesin motors by viral structural proteins (Figure 1)

In the case of HIV-1, the Gag polyprotein directly binds to KIF4 via its matrix (MA) domain, bypassing the need for adaptor mimicry (29). This represents a distinct direct mechanism in which the major structural protein of the virus itself serves as the motor-binding entity. Unlike F12L in VACV, which mimics an adaptor, Gag engages the motor directly, though whether this involves a canonical binding interface comparable to known cargo adaptors remains to be structurally resolved.

### Indirect dependency on constitutive host kinesin trafficking (Figure 2)

As detailed in Section 3.4, HBV represents a distinct category in which the virus does not encode any kinesin-interacting protein but instead relies on the normal physiological function of KIF4A in NTCP trafficking. This indirect mode of host dependency is mechanistically distinct and may be more widespread than currently appreciated: any virus that depends on cell-surface receptors subject to kinesin-mediated trafficking may be vulnerable to perturbation of those transport pathways, even in the absence of direct viral–motor interactions.

### Regulation of motor activity through kinase signaling

Viral kinases (e.g., HSV-1 US3, HCMV UL97) phosphorylate both viral and host substrates that regulate motor function.^27^ Additionally, viruses activate host kinase cascades (MAPK, Src-family kinases, PKC) that can modulate kinesin activity, KLC phosphorylation, and cargo-motor coupling. The phosphorylation of KLC by various kinases regulates cargo release, and viruses may exploit this mechanism to achieve spatiotemporally controlled delivery of viral components.

## Therapeutic targeting of kinesin-dependent viral transport

### Rationale for host-directed antiviral strategies

The deep dependence of viruses on host kinesin motors presents an attractive therapeutic paradigm. Unlike viral targets, host factors are not subject to the rapid mutational escape that limits direct-acting antivirals. Furthermore, because multiple viral families share dependence on the same kinesin pathways, host-directed therapies could potentially provide broad-spectrum antiviral activity. However, the essential nature of kinesin function in uninfected cells raises significant concerns regarding the therapeutic index and toxicity.

### Current approaches

#### 1 Specific kinesin inhibitors

Small-molecule inhibitors of specific kinesin family members have been developed primarily in the context of oncology. Inhibitors of kinesin-5 (Eg5), such as monastrol and ispinesib, disrupt mitotic spindle formation and are currently in clinical trials as anticancer agents.^34^ Their antiviral potential has not been systematically evaluated but could be relevant for viruses that depend on mitotic kinesin functions.

The development of specific inhibitors for transport kinesins (kinesin-1, kinesin-3) is less advanced but represents a promising direction. The structural differences between kinesin family members, particularly in the loop L5 region of the motor domain and in the cargo-binding tail domains, provide opportunities for selective targeting.

#### 2 Disruption of virus–kinesin interfaces

The most promising strategy for achieving antiviral specificity is to target the interface between viral proteins and kinesin motors or their adaptors, rather than inhibiting kinesin function globally. The identification of the molecular determinants governing the kinesin/cargo interaction may help in the design of small molecules that preferentially dock into interaction sites and block transport.

Peptide or small-molecule inhibitors that specifically block, for example, the F12L–KLC interaction in vaccinia virus or the Gag–Rab11 interaction in HIV-1 could provide antiviral activity without disrupting endogenous transport.

### Challenges and future directions

The central challenge in targeting kinesin-dependent viral transport is achieving therapeutic specificity—inhibiting viral exploitation of kinesins while preserving essential cellular transport. This requires detailed structural and mechanistic understanding of virus-specific motor engagement strategies. Advances in cryo-EM, proximity labeling (BioID, APEX), live-cell single-molecule imaging, and optogenetic motor control are providing increasingly precise tools to dissect these interactions.

The KIF4A–NTCP–HBV system exemplifies the therapeutic potential of targeting host transport machinery: Aly et al. demonstrated that KIF4A regulates the anterograde transport of NTCP to the cell surface, and that disruption of this transport pathway—either through KIF4A silencing or pharmacological intervention with the RXR agonist bexarotene and S169—significantly suppresses HBV entry.^9,10^ Notably, bexarotene is an FDA-approved drug, suggesting that repurposing existing compounds to disrupt kinesin-dependent receptor trafficking may represent a viable antiviral strategy with a defined safety profile.

## Limitations and remaining questions

### Current limitations

Despite significant progress, several important limitations in our understanding must be acknowledged. First, much of what is known about kinesin exploitation by viruses derives from studies in transformed cell lines or overexpression systems, which may not faithfully recapitulate the transport dynamics in primary, physiologically-relevant cell types. For example, the role of KIF4A in NTCP trafficking has been characterized primarily in HepG2-NTCP and HuH7-NTCP cell lines; whether the same dependency holds in primary human hepatocytes or in the context of chronic HBV infection *in vivo* remains to be established.

Second, most studies have examined kinesin exploitation in isolation. Given that multiple kinesin families may act redundantly (as suggested by herpesvirus motor redundancy), single-motor perturbation experiments may systematically underestimate the extent and complexity of kinesin dependency networks.

### Key open questions

Several fundamental questions remain unresolved. (i) What is the structural basis for the direct interaction between viral proteins (e.g., Gag MA domain) and kinesin motors, and how does this compare to canonical cargo–adaptor interfaces? High-resolution structural studies of viral protein–kinesin complexes are needed to address this gap. (ii) How do viruses coordinate the switch between dynein-dependent inward transport (during entry) and kinesin-dependent outward transport (during egress)? The molecular mechanism of the “directional switch” in herpesviruses, and whether analogous mechanisms operate in other viruses, remains incompletely resolved. (iii) For indirect kinesin dependencies such as the KIF4A–NTCP axis in HBV, are there analogous receptor-trafficking dependencies for other hepatotropic or tissue-tropic viruses? Systematic screening of virus–receptor trafficking pathways could uncover a broader class of indirect kinesin vulnerabilities. (iv) Can virus-induced remodeling of the tubulin code influence kinesin motor selectivity and thereby enhance specific viral transport pathways? The extent to which viruses manipulate post-translational tubulin modifications to favor their own transport remains largely unexplored. (v) What is the therapeutic window for targeting kinesin-dependent receptor trafficking *in vivo*? Although RXR agonists such as bexarotene show promise *in vitro*, their systemic suppression of KIF4A-dependent transport could have pleiotropic effects on normal hepatocyte physiology, and careful dose–response and toxicity studies in relevant animal models are required.

### Emerging areas

The kinesin dependencies of several important viral pathogens remain entirely uncharacterized. For RNA viruses that form cytoplasmic replication compartments (e.g., flaviviruses, coronaviruses), the contribution of kinesin-mediated transport to the establishment and maintenance of these membranous replication organelles has not been systematically investigated. Similarly, the role of kinesin-14 family members—which mediate minus-end-directed transport and are therefore functionally analogous to cytoplasmic dynein—in viral trafficking has received virtually no attention.

## Conclusions and future perspectives

The kinesin superfamily represents a major, systematically underexplored class of host factors in virology. Herpesvirus axonal transport and vaccinia virus egress have been studied in-depth as paradigms of direct exploitation; however, the KIF4A–NTCP–HBV axis has established indirect host dependency as an equally important and therapeutically tractable mechanism. The kinesin dependencies of most viral families, however, remain poorly characterized, and important questions remain unresolved regarding structural mechanisms, directional switching, and *in vivo* relevance.

The coming decade promises rapid advances in this field, driven by technological developments in live-cell imaging, structural biology, and high-throughput functional genomics. A comprehensive understanding of kinesin exploitation by viruses will not only illuminate fundamental aspects of the host–pathogen relationship but may also yield a new class of broad-spectrum, host-directed, antiviral therapeutics.

## Funding

This study was supported by Grants-in-aid for Scientific Research (Grant number: 23K06575), the Research Program on Hepatitis B from AMED (Grant numbers: 26fk0310532s0102 and 26fk0310528s0102), and the Research Project for Overcoming Hepatitis from AMED (Grant numbers: 26fk0210175h0002 and 26fk0210203h0001).

## Figures and Tables

**Figure 1:**
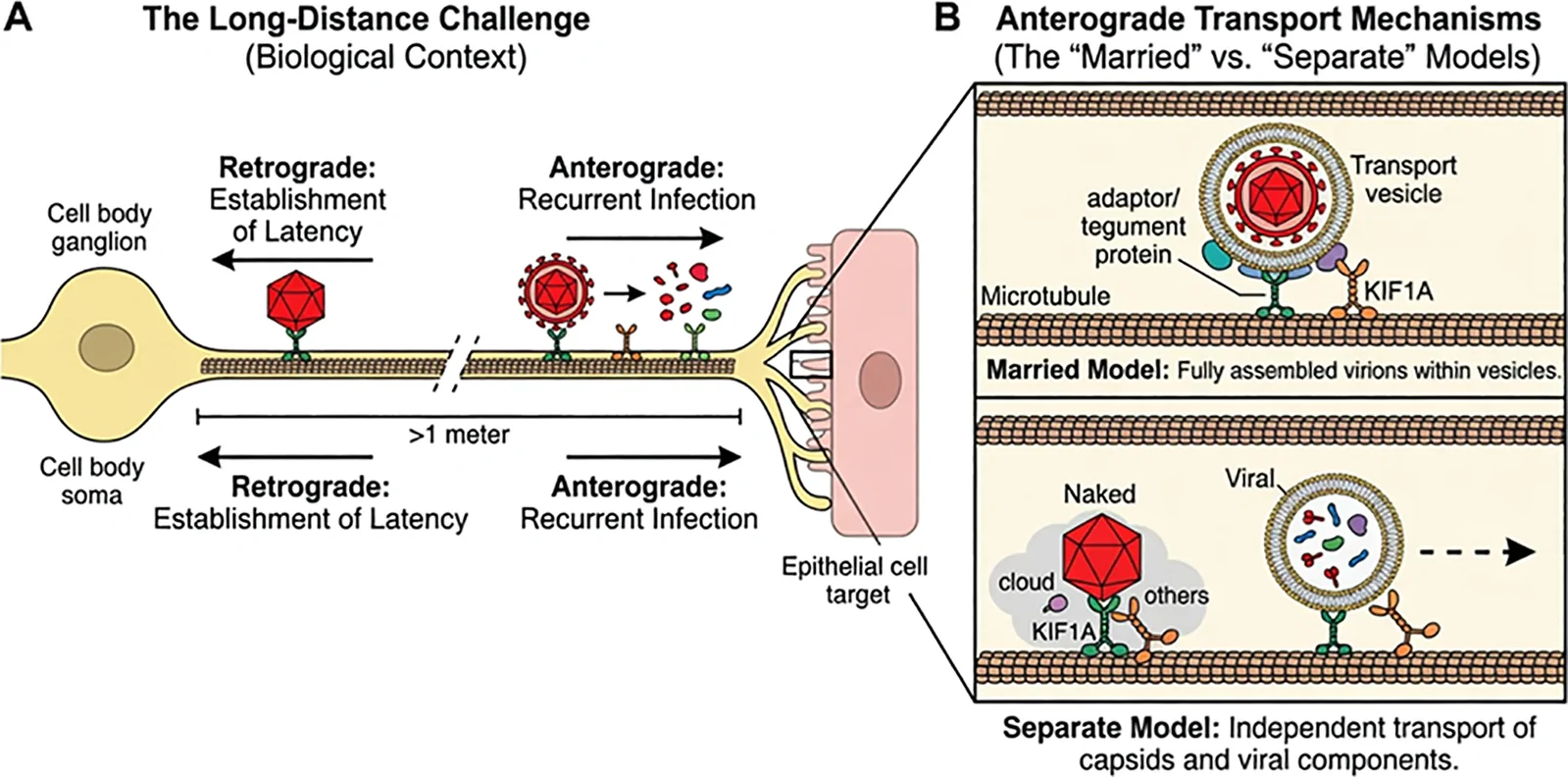
Axonal transport paradigms of alphaherpesviruses (direct kinesin interaction) **(A) The Long-Distance Challenge (Biological Context).** Schematic illustration of the alphaherpesvirus lifecycle in sensory neurons, spanning distances greater than 1 meter. Following initial infection of epithelial cell targets, viral particles undergo retrograde axonal transport toward the neuronal cell body and ganglia, where latency is established. During reactivation, virions are transported anterogradely back to epithelial tissues to establish recurrent infection. Both retrograde and anterograde transport pathways are essential for the herpesvirus lifecycle and neuropathogenesis. **(B) Anterograde Transport Mechanisms: “Married” versus “Separate” Models.** Two competing mechanistic models for alphaherpesvirus anterograde transport are illustrated. In the Married Model (top panel), fully assembled virions are enveloped within membrane-bound transport vesicles and recruited to microtubules through adaptor/tegument proteins that interact with the kinesin motor protein KIF1A. In the Separate Model (bottom panel), viral capsids and other viral components are transported independently as “naked” particles or within distinct vesicles, with their assembly occurring after axonal delivery. This distinction has critical implications for understanding viral egress, neuronal pathogenesis, and the mechanisms underlying recurrent infection.

**Figure 2:**
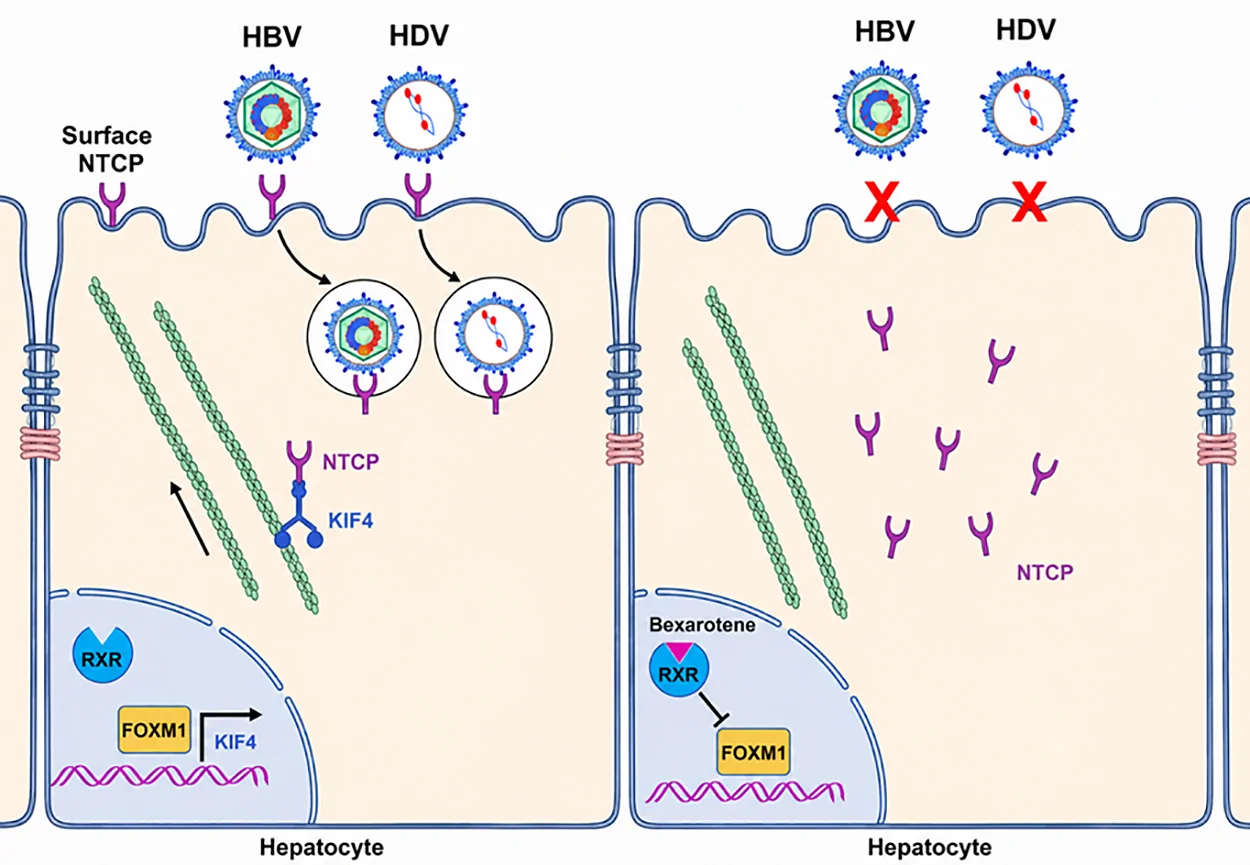
KIF4A as a regulator of HBV entry (indirect dependency on constitutive host kinesin trafficking) Left: FOXM1 activates KIF4 expression, enabling cytoplasmic KIF4 to transport NTCP via microtubules to the hepatocyte surface, facilitating HBV/HDV entry. Right: The RXR agonist Bexarotene suppresses FOXM1 and KIF4, reducing NTCP surface transport and blocking HBV/HDV entry into human hepatocytes.

**Table 1:** The 14 kinesin families and their primary cellular functions

Family	Key Members	Primary Functions	Known Viral Associations
Kinesin-1	KIF5A/B/C	Long-range organelle/vesicle transport	**KIF5:** **HSV-1, PRV**
Kinesin-2	KIF3A/B, KIF17	Intraflagellar transport, Golgi trafficking	**KIF3: HSV-1, PRV**
Kinesin-3	KIF1A/B, KIF13, KIF16B	Synaptic vesicle transport, endosome trafficking	**KIF1A: HSV-1**
Kinesin-4	KIF4A/B, KIF7, KIF21A/B	Chromosome condensation, MT dynamics regulation	**KIF4A: HBV, HIV-1**
Kinesin-5	KIF11/Eg5	Bipolar spindle formation	Not yet described
Kinesin-6	KIF20A/B, KIF23	Cytokinesis, midbody formation	**KIF-23: HIV-1**
Kinesin-7	CENP-E	Kinetochore-MT attachment	Not yet described
Kinesin-8	KIF18A/B	MT length regulation, chromosome alignment	Not yet described
Kinesin-9	KIF6, KIF9	Flagellar motility	Not yet described
Kinesin-10	KIF22	Chromosome arm movement	Not yet described
Kinesin-11	KIF26A/B	Kidney development	Not yet described
Kinesin-12	KIF12, KIF15	Spindle assembly, neuronal polarity	Not yet described
Kinesin-13	KIF2A/B/C	MT depolymerization	Not yet described
Kinesin-14	KIFC1/2/3	Minus-end-directed transport, spindle organization	Not yet described

Abbreviations: HSV-1, herpes simplex virus type 1; PRV, pseudorabies virus; HBV, hepatitis B virus; MT, microtubule.

## References

[1] Luby-Phelps K. Cytoarchitecture and physical properties of cytoplasm: volume, viscosity, diffusion, intracellular surface area. Int Rev Cytol 2000; 192: 189–221. doi:10.1016/s0074-7696(08)60527-6 10.1016/s0074-7696(08)60527-6 10553280

[2] Dodding MP, Way M. Coupling viruses to dynein and kinesin-1. EMBO J 2011; 30: 3527–3539. doi:10.1038/emboj.2011.283 10.1038/emboj.2011.283 PMC3181490 21878994

[3] Mercer J, Helenius A. Virus entry by macropinocytosis. Nat Cell Biol 2009; 11: 510–520. doi:10.1038/ncb0509-510 10.1038/ncb0509-510 19404330

[4] Kardon JR, Vale RD. Regulators of the cytoplasmic dynein motor. Nat Rev Mol Cell Biol 2009; 10: 854–865. doi:10.1038/nrm2804 10.1038/nrm2804 PMC3394690 19935668

[5] Hirokawa N, Noda Y, Tanaka Y, Niwa S. Kinesin superfamily motor proteins and intracellular transport. Nat Rev Mol Cell Biol 2009; 10: 682–696. doi:10.1038/nrm2774 10.1038/nrm2774 19773780

[6] Merino-Gracia J, Garcia-Mayoral MF, Rodriguez-Crespo I. The association of viral proteins with host cell dynein components during virus infection. FEBS J 2011; 278: 2997–3011. doi:10.1111/j.1742-4658.2011.08252.x 10.1111/j.1742-4658.2011.08252.x PMC7164101 21777384

[7] Dohner K, Nagel CH, Sodeik B. Viral stop-and-go along microtubules: taking a ride with dynein and kinesins. Trends Microbiol 2005; 13: 320–327. doi:10.1016/j.tim.2005.05.010 10.1016/j.tim.2005.05.010 15950476

[8] Lawrence CJ, Dawe RK, Christie KR, et al. A standardized kinesin nomenclature. J Cell Biol 2004; 167: 19–22. doi:10.1083/jcb.200408113 10.1083/jcb.200408113 PMC2041940 15479732

[9] Gad SA, Sugiyama M, Tsuge M, et al. The kinesin KIF4 mediates HBV/HDV entry through the regulation of surface NTCP localization and can be targeted by RXR agonists in vitro. PLoS Pathog 2022; 18: e1009983. doi:10.1371/journal.ppat.1009983 10.1371/journal.ppat.1009983 PMC8970526 35312737

[10] Gad SA, Abo Elwafa DA, Hussein SA, Fukano K, Kato T, Shoji I, Chayama K, Muramatsu M, Isogawa M, Wakita T, Merk D, Aly HH. RXR agonist S169 inhibits HBV/HDV entry in vitro by disrupting KIF4-dependent NTCP trafficking. Antiviral Res 2025; 240: 106214. doi:10.1016/j.antiviral.2025.106214 10.1016/j.antiviral.2025.106214 40523562

[11] Vale RD, Milligan RA. The way things move: looking under the hood of molecular motor proteins. Science 2000; 288: 88–95. doi:10.1126/science.288.5463.88 10.1126/science.288.5463.88 10753125

[12] Diwaker D, Kim D, Cordova-Martinez D, Pujari N, Jordan BA, Smith GA, Wilson DW. The gE/gI complex is necessary for kinesin-1 recruitment during alphaherpesvirus egress from neurons. J Virol 2025; 99: e0165024. doi:10.1128/jvi.01650-24 10.1128/jvi.01650-24 PMC11784224 39651860

[13] DuRaine G, Wisner TW, Howard P, Johnson DC. Kinesin-1 Proteins KIF5A, -5B, and -5C Promote Anterograde Transport of Herpes Simplex Virus Enveloped Virions in Axons. J Virol 2018; 92: e01269-18. doi:10.1128/JVI.01269-18 10.1128/JVI.01269-18 PMC6158419 30068641

[14] Gaudin R, de Alencar BC, Jouve M, Bèrre S, Le Bouder E, Schindler M, Varthaman A, Gobert FX, Benaroch P. Critical role for the kinesin KIF3A in the HIV life cycle in primary human macrophages. J Cell Biol 2012; 199: 467–479. doi:10.1083/jcb.201201144 10.1083/jcb.201201144 PMC3483138 23091068

[15] Martinez NW, Xue X, Berro RG, Kreitzer G, Resh MD. Kinesin KIF4 regulates intracellular trafficking and stability of the human immunodeficiency virus type 1 Gag polyprotein. J Virol 2008; 82: 9937–9950. doi:10.1128/JVI.00819-08 10.1128/JVI.00819-08 PMC2566262 18684836

[16] Pernigo S, Lamprecht A, Steiner RA, Dodding MP. Structural basis for kinesin-1:cargo recognition. Science 2013; 340: 356–359. doi:10.1126/science.1234264 10.1126/science.1234264 PMC3693442 23519214

[17] Verhey KJ, Hammond JW. Traffic control: regulation of kinesin motors. Nat Rev Mol Cell Biol 2009; 10: 765–777. doi:10.1038/nrm2782 10.1038/nrm2782 19851335

[18] Bowman AB, Kamal A, Ritchings BW, Philp AV, McGrail M, Gindhart JG, Goldstein LS. Kinesin-dependent axonal transport is mediated by the sunday driver (SYD) protein. Cell 2000; 103: 583–594. doi:10.1016/s0092-8674(00)00162-8 10.1016/s0092-8674(00)00162-8 11106729

[19] Coy DL, Hancock WO, Wagenbach M, Howard J. Kinesin’s tail domain is an inhibitory regulator of the motor domain. Nat Cell Biol 1999; 1: 288–292. doi:10.1038/13001 10.1038/13001 10559941

[20] Janke C, Magiera MM. The tubulin code and its role in controlling microtubule properties and functions. Nat Rev Mol Cell Biol 2020; 21: 307–326. doi:10.1038/s41580-020-0214-3 10.1038/s41580-020-0214-3 32107477

[21] Antinone SE, Smith GA. Retrograde axon transport of herpes simplex virus and pseudorabies virus: a live-cell comparative analysis. J Virol 2010; 84: 1504–1512. doi:10.1128/JVI.02029-09 10.1128/JVI.02029-09 PMC2812336 19923187

[22] DuRaine G, Johnson DC. Anterograde transport of alpha-herpesviruses in neuronal axons. Virology 2021; 559: 65–73. doi:10.1016/j.virol.2021.02.011 10.1016/j.virol.2021.02.011 33836340

[23] Kramer T, Greco TM, Taylor MP, Ambrosini AE, Cristea IM, Enquist LW. Kinesin-3 mediates axonal sorting and directional transport of alphaherpesvirus particles in neurons. Cell Host Microbe 2012; 12: 806–814. doi:10.1016/j.chom.2012.10.013 10.1016/j.chom.2012.10.013 PMC3527838 23245325

[24] Radtke K, Kieneke D, Wolfstein A, Michael K, Steffen W, Scholz T, Karger A, Sodeik B. Plus- and minus-end directed microtubule motors bind simultaneously to herpes simplex virus capsids using different inner tegument structures. PLoS Pathog 2010; 6: e1000991. doi:10.1371/journal.ppat.1000991 10.1371/journal.ppat.1000991 PMC2900298 20628567

[25] Zaichick SV, Bohannon KP, Hughes A, Sollars PJ, Pickard GE, Smith GA. The herpesvirus VP1/2 protein is an effector of dynein-mediated capsid transport and neuroinvasion. Cell Host Microbe 2013; 13: 193–203. doi:10.1016/j.chom.2013.01.009 10.1016/j.chom.2013.01.009 PMC3808164 23414759

[26] Scherer J, Hogue IB, Yaffe ZA, Tanneti NS, Winer BY, Vershinin M, Enquist LW. A kinesin-3 recruitment complex facilitates axonal sorting of enveloped alpha herpesvirus capsids. PLoS Pathog 2020; 16: e1007985. doi:10.1371/journal.ppat.1007985 10.1371/journal.ppat.1007985 PMC7010296 31995633

[27] Xiong R, Rao P, Kim S, Li M, Wen X, Yuan W. Herpes Simplex Virus 1 US3 Phosphorylates Cellular KIF3A To Downregulate CD1d Expression. J Virol 2015; 89: 6646–6655. doi:10.1128/JVI.00214-15 10.1128/JVI.00214-15 PMC4468489 25878107

[28] McDonald D, Vodicka MA, Lucero G, Svitkina TM, Borisy GG, Emerman M, Hope TJ. Visualization of the intracellular behavior of HIV in living cells. J Cell Biol 2002; 159: 441–452. doi:10.1083/jcb.200203150 10.1083/jcb.200203150 PMC2173076 12417576

[29] Tang Y, Winkler U, Freed EO, Torrey TA, Kim W, Li H, Goff SP, Morse HC 3rd. Cellular motor protein KIF-4 associates with retroviral Gag. J Virol 1999; 73: 10508–10513. doi:10.1128/JVI.73.12.10508-10513.1999 10.1128/jvi.73.12.10508-10513.1999 PMC113106 10559369

[30] Smith GL, Vanderplasschen A, Law M. The formation and function of extracellular enveloped vaccinia virus. J Gen Virol 2002; 83: 2915–2931. doi:10.1099/0022-1317-83-12-2915 10.1099/0022-1317-83-12-2915 12466468

[31] Rietdorf J, Ploubidou A, Reckmann I, Holmström A, Frischknecht F, Zettl M, Zimmermann T, Way M. Kinesin-dependent movement on microtubules precedes actin-based motility of vaccinia virus. Nat Cell Biol 2001; 3: 992–1000. doi:10.1038/ncb1101-992 10.1038/ncb1101-992 11715020

[32] Frischknecht F, Moreau V, Röttger S, Gonfloni S, Reckmann I, Superti-Furga G, Way M. Actin-based motility of vaccinia virus mimics receptor tyrosine kinase signalling. Nature 1999; 401: 926–929. doi:10.1038/44860 10.1038/44860 10553910

[33] Ward BM, Moss B. Visualization of intracellular movement of vaccinia virus virions containing a green fluorescent protein-B5R membrane protein chimera. J Virol 2001; 75: 4802–4813. doi:10.1128/JVI.75.10.4802-4813.2001 10.1128/JVI.75.10.4802-4813.2001 PMC114235 11312352

[34] Welte MA. Bidirectional transport along microtubules. Curr Biol 2004; 14: R525–R537. doi:10.1016/j.cub.2004.06.045 10.1016/j.cub.2004.06.045 15242636

